# Acupuncture at the P6 Acupoint to Prevent Postoperative Pain after Craniotomy: A Randomized, Placebo-Controlled Study

**DOI:** 10.1155/2021/6619855

**Published:** 2021-03-17

**Authors:** Jian-Qin Lv, Peng-Cheng Li, Li Zhou, Wen-Fu Tang, Ning Li

**Affiliations:** ^1^Department of Integrative Medicine, West China Hospital, Sichuan University, Chengdu, China; ^2^Department of Neurosurgery, West China Hospital, Sichuan University, Chengdu, China; ^3^Department of Anesthesiology, West China Hospital, Sichuan University, Chengdu, China

## Abstract

**Objective:**

Acute pain management after craniotomy can be challenging. Previous studies have shown inadequate pain control following the procedure. Oral medication can sometimes be delayed by postoperative nausea, and use of anesthetics may impair the assessment of brain function. We conducted this prospective study to evaluate the effect of acupuncture at the P6 acupoint on postoperative pain, nausea, and vomiting in patients undergoing craniotomy.

**Methods:**

The authors conducted a randomized, placebo-controlled trial among 120 patients scheduled for craniotomy under general anesthesia. 120 patients were randomly assigned into an acupuncture group or a sham acupuncture group. All patients received standardized anesthesia and analgesia treatment. Acupuncture was executed in the recovery room after surgery. For the acupuncture group, the P6 points on each wrist were punctured perpendicularly to a depth of 20 mm. Needles were retained for 30 min and stimulated every 10 min to maintain the De-Qi sensation. For the sham acupuncture group, sham points on each wrist were punctured perpendicularly to a depth of 5 mm. Needles were retained for 30 min with no stimulation during the duration. The postoperative pain scores, PONV, and dose of tramadol were assessed 24 h, 48 h, and 72 h after surgery.

**Results:**

A total of 117 patients completed the study. There was no statistically significant difference in baseline data between the two groups (*P* > 0.05). The VAS pain score of the acupuncture group was lower than that of the sham acupuncture group, and this difference was statistically significant (*P*=0.002). There was no difference in pain scores between the two groups during 0–24 h and 48–72 h (*P* > 0.05). The incidence of vomiting in the acupuncture group was lower than that in the sham acupuncture group during the 0–24 h period (13.8% vs. 28.8%, *P*=0.048). There was no difference in vomiting, however, during the 24–72 h period (*P* > 0.05). No significant differences were found in the degree of nausea and the dose of tramadol between the two groups at either time point in the acupuncture group and sham acupuncture group.

**Conclusion:**

The use of acupuncture at the P6 acupoint in neurosurgery patients did result in significantly lower pain scores and reduction in the incidence of vomiting after craniotomy. There were no significant side effects. Acupuncture at the P6 acupoint was well tolerated and safe in this patient population.

## 1. Introduction

A review paper has analyzed publications in the international literature regarding the problem of acute postoperative pain in neurosurgical patients who have undergone craniotomy. This review indicated that the problem of acute postoperative pain in patients after craniotomy has been previously underestimated. It had been mistakenly thought that these patients do not experience any pain in the early postoperative period. Results of recent studies have shown that up to 80% of these patients may in fact experience acute mild to severe pain [1]. Postoperative pain after craniotomy is unfavorable for the recovery of patients. Pain can cause postoperative complications including anxiety, nausea, vomiting, arterial hypertension, intracranial hypertension, and postoperative hemorrhage [2, 3].

Pain management after craniotomy is a clinical problem. The main challenge is that analgesic therapy may interfere with nervous system function and postoperative evaluation. Numerous studies have shown that patients in the neurocritical care (NCC) unit experience inadequate pain control [4, 5]. Optimizing patient comfort after craniotomy is often difficult because the use of narcotic medications can impair the clinical evaluation of neurological function. The sedation and pupillary miosis caused by opioids can directly mask seminal signs of intracranial pathology, and thus, opioids are used judiciously. Additionally, craniotomy is associated with postoperative nausea, which delays the use of oral medications.

Multimodal analgesia is advocated currently for postoperative pain. There are two aspects of multimodal analgesia. (1) Balanced anesthesia affects the local anesthetics of the scalp, opioid, and nonopioid analgesics. (2) Nonpharmacologic treatments include distractibility, massage therapy, transcutaneous electrical nerve stimulation therapy, and acupuncture.

Acupuncture is a traditional Chinese medical technique that has been extensively used as a nonpharmacological analgesic therapy since being developed 2500 years ago. Over the last decade, many clinical studies have focused on acupuncture in the treatment of postoperative pain. For example, a systematic review suggested that acupuncture can relieve acute postoperative pain after back surgery [6]. Another study suggested that acupuncture may help reduce pain during panretinal photocoagulation treatment [7]. Several clinical studies have shown that acupuncture may be effective in improving postoperative analgesia, reducing intraoperative anesthetic requirements and immunosuppression, and reducing the incidence of anesthesia-related side effects [8, 9].

Stimulation of specific points, by using needles or electrodes, releases neurochemical substrates that may block the incoming pain information. Acupuncture can raise the pain threshold. It also reduces pain intensity [10]. The P6 acupoint is one of the most commonly used and well-investigated acupoints for PONV prophylaxis and postoperative pain treatment. Based on the theory of meridian and evidence from previous studies [6, 9], we chose the P6 acupoint for treatment.

The patient's awareness assessment will not be affected because acupuncture is a nonsedating treatment. The aim of this controlled, randomized, single-blind study was to evaluate the effect of acupuncture at the P6 acupoint on postoperative pain following craniotomy. We hypothesized that acupuncture at the P6 acupoint may reduce the postoperative pain experienced by patients.

## 2. Materials and Methods

### 2.1. Study Design and Oversight

Our study protocol was published in Trails in 2013. We followed the methods of Lv et al. [11]. This randomized, blind, controlled trial was approved by the ethics review board of West China Hospital, Sichuan University, and registered with the Chinese Clinical Trial Registry (registration number: ChiCTR-TRC-13003026). All authors assume responsibility for the accuracy and completeness of the data and analyses.

### 2.2. Study Population

All participants gave their written informed consent before being enrolled in this study. In this prospective randomized controlled trial, all cases were from the Department of Neurosurgery, West China Hospital, Sichuan University. Patients were enrolled in the study from October 2014 to September 2017. A total of 120 patients were recruited. SAS 11.0 statistical software was used to design the random number. Patients were randomly assigned into either an acupuncture group or a sham acupuncture group. All the subjects involved were unaware to which group they had been assigned, as was the doctor who observed the postoperative pain and other outcomes.

### 2.3. Inclusion Criteria

Patients who fulfilled the following conditions were included: (1) scheduled for neurosurgery requiring opening of the cranium and dura; (2) aged between 18 and 70 years old; (3) the American Society of Anesthesiologists (ASA) physical status classification of I or II; (4) undergoing general anesthesia; (5) no history of PONV or motion sickness; (6) no use of antiemetics and analgesia 24 hours before surgery; (7) willing to participate; (8) no experience with acupuncture; and (9) having signed an informed consent form.

### 2.4. Exclusion Criteria

Participants that met any of the following criteria were excluded: (1) pain 24 hours before surgery; (2) pregnant or lactating women; (3) drug or alcohol abusers; (4) recipients of chemotherapy or radiation therapy during the previous 7 days; (5) having a cardiac pacemaker fitted; (6) menstruating phase of the menstrual cycle; (7) refusal to accept acupuncture treatment; (8) mental disorder; (9) history of epilepsy and still taking an antiepileptic medicine; (10) unconscious before the surgery; (11) cannot normally communicate; (12) undergoing ventricle or brainstem surgery; (13) cerebral perfusion pressure (CPP) of less than 50 mmHg or greater than 150 mmHg; (14) poorly controlled diabetes mellitus (fasting plasma glucose greater than 12 mmol/L); (15) bleeding disorders (hemophilia or afibrinogenemia); and (16) serious systemic disease (AIDS or sepsis).

### 2.5. Dropout Criteria

Participants who met any of the following criteria were withdrawn from the study: (1) death; (2) waking more than 2 hours after surgery; (3) trachea intubation; (4) persistent coma; (5) cognitive impairment; and (6) further surgery or transfer to the ICU if necessary for the aggravation of the disease. Patients who were withdrawn were not replaced.

### 2.6. Anesthesia and Postoperative Analgesia

All patients underwent general anesthesia with endotracheal intubation. Blood pressure, heart rate, pulse oximetry, and end tidal CO_2_ were routinely monitored. Induction of anesthesia was achieved with midazolam 0.05 mg/kg, sufentanil 0.3 *μ*g/kg, atracurium 0.15 mg/kg, and propofol 2 mg/kg. When endotracheal intubation and gastrointestinal decompression with either an orogastric or nasogastric tube were undertaken, the anesthesia was maintained with 50% nitrous oxide and 3% sevoflurane. The concentration of sevoflurane was adjusted according to BIS and the vital signs; if hypotension occurred and the BIS was low, the sevoflurane dosage was decreased. After the operation had commenced, participants were given sufentanil 0.2 *μ*g/kg and atracurium 0.1 mg/kg intermittently. 30 minutes prior to the end of the operation, the patients were treated with prophylactic antiemetic drugs: ondansetron injection 8 mg according to the advice of doctors. After surgery, patients were continually monitored in the postanesthesia care unit (PACU) with continued ventilator support. The tracheal tube was removed after the patients woke. The time from the start of anesthesia induction to the time of removal of the tube was recorded. Patients who then met the criteria (Steward Rating Scale ≥4 and the blood gas index of special patients being normal as judged by the anesthetist) were sent back to the ward.

After the participants returned to the neurosurgical ward, the doctor decided whether to give pain medication according to the degree of the patient's pain. The patient was given a 10 mg intramuscular injection of tramadol if necessary, and the degree of postoperative pain was recorded when the patient was given the drug.

Randomization and blinding SAS 11.0 statistical software was used to design the random number of patients for the study. The included participants were randomly enrolled by sealed envelope and assigned to the acupuncture group or the sham acupuncture group. Patient allocations were performed by a clinical assistant trained in institutional review board policies. Patients in the two acupuncture groups were unaware to which acupuncture group they were assigned. The outcome assessors, data collectors, and statisticians were also blinded to group allocations during the study.

### 2.7. Interventions

For the acupuncture group, after skin cleaning with 75% alcohol swab, sterile and disposable stainless steel needles (Wuxi Jiajian; 0.25 × 25 mm; made in Jiangsu, China) are quickly and perpendicularly inserted into the skin at P6 acupoints bilaterally to a depth of 20 mm. In this group, downward pressure and upward lifting combined with twirling the needle was used to achieve De-Qi sensation (sensation of soreness, numbness, distention, or radiating, which is considered to indicate effective needling). The needles were kept in place for 30 min and manipulated manually every 10 min to maintain the De-Qi sensation. When the treatment period was over, all needles were carefully removed, and the puncture sites were covered with sterile swabs to avoid bleeding. Acupuncture was performed by licensed acupuncturists with more than 5 years of experience.

**Figure 1:**
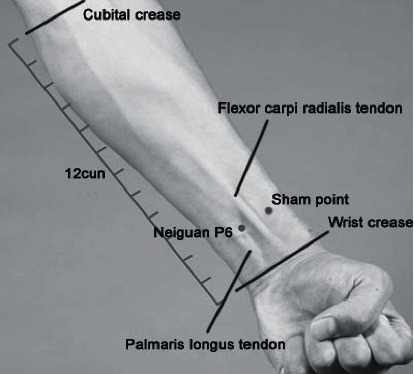
Location of the P6 acupoint and sham acupoint.

For the sham acupuncture group, sham points, which are superficial, nonacupoints at the radial side of each wrist, 15 mm away from each P6 acupoint, were used (Figure 1). After skin cleaning with 75% alcohol swab, sterile and disposable stainless steel needles (Wuxi Jiajian; 0.25 × 25 mm; made in Jiangsu, China) were quickly and perpendicularly inserted into the skin at sham acupoints bilaterally to a depth of 5 mm. The De-Qi sensation was not required in this group. The needles were retained for 30 min as with the acupuncture group, but there was no stimulation or manipulation of the needles. After 30 min, the same method to remove the needles in the acupuncture group was used. Acupuncture was performed by licensed acupuncturists with more than 5 years of experience.

Figure 1 is reproduced from Lv et al., P6 acupoint stimulation for prevention of postoperative nausea and vomiting in patients undergoing craniotomy: study protocol for a randomized controlled trial, BMC, 2013 (under the Creative Commons Attribution License/public domain).

### 2.8. Measures

The study period covered 72 h after surgery. A separate research nurse, not involved in the management of patients, recorded anesthesia time, surgery time, endotracheal intubation time, patient demographics, and preoperative data for each patient. Demographic and preoperative data included the following: age, gender, weight, acupuncture experience, and smoking history. Another blinded observer (nurse) recorded the postoperative data, which included assessing the postoperative pain score at rest. Pain scores were collected prospectively during the 72 h postoperative period using the Visual Analog Scale (VAS). Patients were asked to rate their pain on a 0–10 scale, where “0” represented “no pain” and “10” represented the “worst pain I have ever experienced.” The data were recorded by the nurse. The physicians recorded the use time and dosage of rescue analgesia (tramadol) for each patient when they requested rescue therapy, and the records were handed over to the observer for assessment. The incidence of postoperative nausea and vomiting was recorded. Assessments were performed at 24, 48, and 72 h. Reasons for withdrawal and acupuncture-associated adverse events (AEs), including bleeding, subcutaneous hemorrhage, hematoma, fainting, serious pain, and local infection, were recorded during the study.

### 2.9. Statistical Analysis

Since there had been no previous studies on acupuncture to prevent postcraniotomy pain, we drew on the results of a similar study that used tramadol. In that study, there was an average VAS score of 3 in the tramadol group compared with 4.7 in the control group [3]. According to the results of that previous study and our pilot study, we anticipated an average VAS score of 2 after acupuncture treatment between the acupuncture group and sham acupuncture group. The sample size was determined by using PASS 15.0 with *α* = 0.05 (two sides) and *β* = 0.01 (power 90%). The resulting sample size is 31 patients per group. Estimating that 20% of patients might be lost means using at least 40 subjects per group. Intending also to observe the occurrence of postoperative nausea and vomiting, we set the sample size of each group at 60 cases in order to ensure statistical viability.

All data input and the statistical analysis were performed in the Department of Epidemiology and Hygienic Statistics of West China Hospital of Sichuan University using SPSS 19.0. Data were checked for normality with the Kolmogorov–Smirnov test. Normally distributed variables were presented as the mean (SD) and were analyzed by a two-sample *t*-test. Nonnormally distributed variables (i.e., pain and PONV) were described as the median (interquartile range (IQR)) and were compared by the Mann–Whitney *U* test. A *P* value of <0.05 was considered statistically significant.

## 3. Results

### 3.1. Participants and Baseline Characteristics

**Figure 2:**
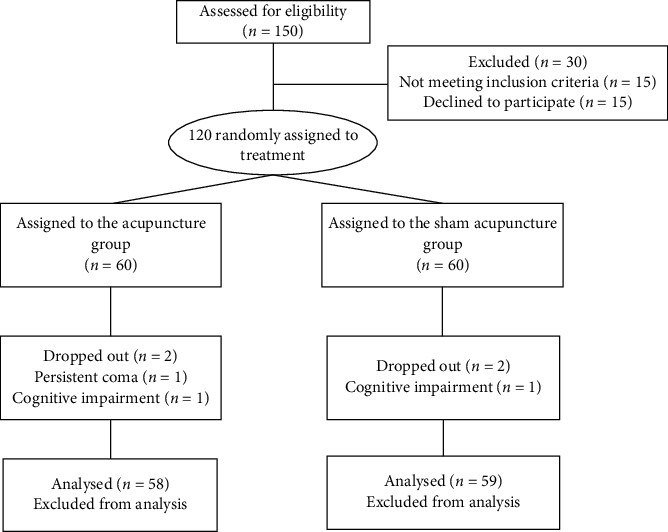
CONSORT flow diagram of the trial.

A total of 120 patients were enrolled in the study. Three of these (2.5%), two in the acupuncture group and one in the sham acupuncture group, were withdrawn after they subsequently met the withdrawal criteria. Two of these suffered persistent coma, the other cognitive impairment. The data of the remaining 117 patients (46 male and 71 female patients) were analyzed. The characteristics of the patients in the two groups—those receiving acupuncture or sham acupuncture—and their previous medical history were not significantly different (*P* > 0.05) (Figure 2 and Table 1).

Figure 2 is reproduced from Lv et al., P6 acupoint stimulation for prevention of postoperative nausea and vomiting in patients undergoing craniotomy: study protocol for a randomized controlled trial, BMC, 2013 (under the Creative Commons Attribution License/public domain).

### 3.2. Effects of Acupuncture at the P6 Acupoint on Postoperative Pain

There was no statistically significant difference between the two groups regarding their pain scores 0–24 h after surgery (*P*=0.064). The VAS pain score of the acupuncture group, however, was lower than that of the sham acupuncture group, and this difference is statistically significant 24–48 h after surgery (*P*=0.002). There was no statistically significant difference in the VAS pain score of the two groups 48–72 h after craniotomy (*P*=0.254). There was no statistically significant difference between the two groups in the analgesic drug remediation at each period after craniotomy (*P* > 0.05) (Tables 2 and 3).

### 3.3. Effects of Acupuncture at the P6 Acupoint on Postoperative Nausea and Vomiting

In the 0–24 h after surgery, the incidence of vomiting in the acupuncture group was lower than that in the sham acupuncture group. There was a statistically significant difference in the incidence of vomiting between the two groups during 24 h following craniotomy (13.8% vs. 28.8%, *P*=0.048), though there was no statistically significant difference in the incidence of vomiting between the two groups during 48–72 h following craniotomy (*P* > 0.05). There was no statistical difference in the degree of nausea between the two groups 0–24 h, 24–48 h, and 48–72 h after surgery (*P* > 0.05) (Tables 4 and 5).

### 3.4. Safety

Two patients (one in each group) reported AEs during the testing period. These patients had subcutaneous hemorrhage. All AEs were reported as mild, and none required special medical intervention. The two patients fully recovered from the AEs and did not withdraw from the trial.

## 4. Discussion

It has been accepted in the past that the pain accompanying intracranial surgery was minimal and, when present, dangerous to treat. As a consequence, analgesic therapy for this group of patients has generally been modest [12]. It is now known that pain from intracranial surgery is comparably similar to that caused by other surgical procedures, and it is becoming acceptable to treat this pain. Using traditionally limited analgesic therapy, 69% of patients undergoing craniotomy report some period of pain that is moderate to severe (pain rating: 4/10) on the first postoperative day, and 48% of patients experience this level of pain during some portion of the second postoperative day [13]. Pain can induce nausea and vomiting and aggravate brain edema. The objective of pain control after craniotomy in the present study is to reduce the use of analgesic and sedative drugs that can cause gastrointestinal reactions and interfere with the consciousness of the patients following surgery.

The results of this study indicate that acupuncture at the P6 acupoint can reduce postoperative pain after craniotomy and may also reduce the incidence of vomiting in patients following craniotomy. This study found that patients had moderate-to-severe pain after the procedure, and the pain is more obvious during the first postoperative 24 hours. In both sham acupuncture and acupuncture groups, the VAS scores were gradually decreased in the 24–48 h period after surgery. This result is similar to a previous study indicating that postoperative pain began to decrease from 24–48 h [14].

The acupuncture and sham acupuncture groups displayed no difference in postoperative pain scores 0–24 h after surgery. A possible explanation for this finding is that intraoperative fentanyl may help to alleviate VAS mainly in the early postoperative period. The VAS pain score in the two groups during 24–48 h after surgery, however, did show significant difference. This result is consistent with the conclusion of Mali's study which indicated that multiple electroacupuncture stimulation at different time points can reduce the VAS pain score of postoperative patients with gastrointestinal tumors within 72 h [15]. This is consistent with Chung et al.'s research. This group demonstrated that combined auricular acupressure and TEAS decreased postoperative pain, the use of analgesic morphine, and morphine-related side effects. IAS provides better analgesia when used in conjunction with PCA after lumbar spine surgery and can be regarded as a component of multimodal analgesia [16]. This result is contrary to An et al. [17], who found that, during the first six hours following surgery, the VAS score of the electric acupuncture group was much lower than that of the control group, whereas there was no difference during 6–48 h in patients undergoing a supratentorial craniotomy. We hypothesized this difference may be attributable to the patient-controlled intravenous analgesia (PCIA) they used in the first 48 h. This may be the case, in our study, where no PCIA was given. Our results that acupuncture can reduce the postoperative pain of craniotomy in 24–48 h contrast with the research of Liu et al. [18], who found that pain scores after supratentorial craniotomy were significantly lower at postoperative day 1 in the TEAS group than in the sham group. However, the VAS pain score was higher in the TEAS group on postoperative days 2 and 3. They concluded the explanation for this is unknown and may relate to the short-term anesthetic effects of TEAS so that the analgesic effect of TEAS may cease after the operation without further stimulation. We speculate that the difference may be associated with the use of analgesic drugs within 72 h (they also used PCIA) after surgery and different stimulation methods (they used TEAS; we used acupuncture). The effect of acupuncture lasts for a prolonged time after the insertion of the needle. The aftereffects are more significant because they are stronger, broader, more lasting, and can accumulate [19]. There was no significant difference in pain scores during 48–72 h between the two groups. We speculate that this may be related to acupuncture intervention time. It had been 48 hours since the acupuncture intervention, and the aftereffects of acupuncture may have dissipated.

Tramadol is a weak *μ*-opioid receptor agonist that releases serotonin and inhibits the reuptake of norepinephrine. It is used for the management of postoperative pain in neurosurgical patients in our institution. Tramadol can provide effective pain relief without the side effects associated with opioids or the inhibitory effects on platelets induced by NSAIDs. Analgesics such as paracetamol and NSAIDs are less frequently used perioperatively to treat postoperative pain in our institution.

This study indicates that acupuncture at the P6 acupoint cannot reduce postoperative analgesic drug (tramadol) use during postoperative 0–72 h. The application rate of analgesic drugs is 8.6% which is much lower than what was found in previous domestic research [20]. The frequently missed diagnosis of postoperative pain and the side effects of analgesic drug use are the main reasons for the low application rate of analgesic drugs [5, 21].

PONV is a common complaint after craniotomy, with an incidence of up to 79% [22]. It can have a highly negative impact on surgical outcomes and is often rated by patients as worse than postoperative pain [23]. Nausea and vomiting after craniotomy also cause electrolyte disorders, intracranial hypertension, and prolonged hospital stays [24].

Our study indicates that acupuncture at the P6 acupoint can reduce postoperative pain, as well as reduce the incidence of vomiting in patients following craniotomy.

Previous studies have shown that nausea and vomiting can also occur within the 24 h postoperative period [17, 25]. This is similar to the results of our study, in which postoperative vomiting occurred in up to 28.8% of patients and vomiting occurred within 24 h. The incidence of vomiting was gradually reduced after 24 h. The results of this study showed that acupuncture can reduce the incidence of vomiting in 24 h following surgery. There was no significant difference between the acupuncture and sham acupuncture groups during 24–72 h after surgery. This agrees with the results of our previous study showing that P6 acupuncture can reduce the degree of postoperative nausea and vomiting [26]. This study also suggested that electrical stimulation at the P6 acupoint can reduce the incidence of nausea and vomiting after craniotomy. It has been suggested that similar effects were observed after treatments involving stimulation at P6 for prevention of postoperative nausea and vomiting [27].

The acupoints and modes used for stimulation may significantly affect the outcome of acupuncture for postoperative nausea and vomiting and postoperative pain relief. In our previous study, we found that P6 acupuncture can reduce the degree of postoperative nausea and postoperative pain [26]. The P6 acupoint is one of the most commonly used and well-investigated acupoints for PONV prophylaxis and postoperative pain treatment. Based on the theory of meridian and evidence from previous studies, we chose the P6 acupoint for treatment.

This is a single-blind, placebo-controlled, randomized trial. The De-Qi response is particularly important. The needle points that readily produce a strong De-Qi sensation are thought to provide better efficacy in patients. Acupuncture stimulation patients were awake during this study, so they were able to confirm the De-Qi sensation. We used shallow skin penetration at nonacupoint caves in the control group so that patients received a sting from the needle, but the possible efficacy of acupuncture was eliminated. The evaluators did not know to which group the patients belonged to. This renders the findings more reliable. However, the degree of pain and nausea evaluation indicators may be dependent on the patient's educational level, and understanding of the degree of pain may affect the results of the study.

Our study has some limitations:

  Patients' preconceived expectations of acupuncture treatment may affect their perceptions regarding the effects of the treatment on their pain and nausea. We should, in further studies, conduct questionnaires asking patients about their expectations of acupuncture treatment.
  Blinding between the two groups was not evaluated by asking participants to guess to which group they were assigned at the end of the intervention.
  We found that there were potential patients unwilling to take part in the study due to their fear of pain caused by acupuncture treatment. Further studies could possibly use more painless treatments such as auricular bean-embedding therapy or intradermal needling.
  There have been very few studies of this surgery type, mainly carried out within the same institution. This makes comparing the results with other independent researchers difficult. Our results will hopefully encourage other groups to carry out similar research.

## 5. Conclusions

Compared with the sham acupuncture group, the true acupuncture patients treated using the P6 acupoint experienced reduced levels of pain after craniotomy during the 24–48 h postoperative period. The incidence of vomiting within the 24 hours following craniotomy was also reduced. In addition, the acupuncture treatment also proved safe for patients.

## Figures and Tables

**Table 1:** Demographic data and surgical characteristics.

Characteristic	Acupuncture group (*N* = 58)	Sham acupuncture group (*N* = 59)	*p* values
Age (years)	47.97 ± 12.69	46.19 ± 14.76	0.486
Height (cm)	162.09 ± 7.88	161.69 ± 7.19	0.779
Weight (kg)	59.50 ± 10.13	63.12 ± 11.85	0.079
Anesthesia time (min)	257.00 ± 92.41	275.75 ± 102.88	0.302
Operation time (min)	166.93 ± 73.34	182.42 ± 91.44	0.315
Dose of sufentanil (µg)	30 (25–30)	30 (25–35)	0.593
Infusion amount (ml)	2471.55 ± 1067.26	2655.42 ± 961.49	0.329
Gender			0.651
Female (%)	34 (58.6)	37 (62.7)	
Male (%)	24 (41.4)	22 (37.3)	
Smoking history			0.781
No (%)	46 (79.3)	48 (81.4)	
Yes (%)	12 (20.7)	11 (18.6)	
The history of surgery			0.949
No (%)	39 (67.2)	40 (67.8)	
Yes (%)	19 (32.8)	19 (32.2)	
Migraine			0.100
No (%)	51 (87.9)	45 (76.3)	
Yes (%)	7 (12.1)	14 (23.7)	
History of opioid use			0.896
No (%)	39 (67.2)	39 (66.1)	
Yes (%)	19 (32.8)	20 (33.9)	
Motion sickness			0.246
No (%)	50 (86.2)	46 (78.0)	
Yes (%)	8 (13.8)	13 (22.0)	
The type of surgery			0.11
Supratentorial (%)	37 (63.8)	29 (49.2)	
Subtentorial (%)	21 (36.2)	30 (50.8)	

**Table 2:** Patient-reported pain scores over time.

	Acupuncture group (*N* = 58)	Sham acupuncture group (*N* = 59)	*p* values
VAS pain score 0–24 h after operation	3 (2–5)	3 (3–5)	0.064
VAS pain score 24–48 h after operation	3 (0–4)	4 (3–5)	0.002
VAS pain score 48–72 h after operation	2 (0–4)	3 (2–3)	0.254

**Table 3:** Postoperative analgesic drug (tramadol) use over time.

	Acupuncture group (*N* = 58)	Sham acupuncture group (*N* = 59)	*p* values
The use of tramadol 0–24 h after operation			0.977
No (%)	53 (91.4)	54 (91.5)	
Yes (%)	5 (8.6)	5 (8.5)	

The use of tramadol 24–48 h after operation			0.623
No (%)	57 (98.3)	56 (94.9)	
Yes (%)	1 (1.7)	3 (5.1)	

The use of tramadol 48–72 h after operation			1.000
No (%)	58 (100)	58 (98.3)	
Yes (%)	0 (0)	1 (1.7)	

**Table 4:** The incidence of vomiting over time.

	Acupuncture group (*N* = 58)	Sham acupuncture group (*N* = 59)	*p* values
Vomiting in 0–24 h after operation			0.048
No (%)	50 (86.2)	42 (71.2)	
Yes (%)	8 (13.8)	17 (28.8)	

Vomiting in 24–48 h after operation			0.969
No (%)	49 (84.5)	50 (84.7)	
Yes (%)	9 (15.5)	9 (15.3)	

Vomiting in 48–72 h after operation			1.000
No (%)	54 (93.1)	54 (91.5)	
Yes (%)	4 (6.9)	5 (8.5)	

**Table 5:** Postoperative nausea over time.

	Acupuncture group (*N* = 58)	Sham acupuncture group (*N* = 59)	*p* values
Score for nausea 0–24 hours after operation	0 (0-1)	0 (0-1)	0.543
Score for nausea 24–48 hours after operation	0 (0-1)	0 (0-0)	0.934
Score for nausea 48–72 hours after operation	0 (0-0)	0 (0-0)	0.822

## Data Availability

The datasets used and/or analyzed will be made available from the corresponding author upon reasonable request.
